# Hydroquinone Exposure Worsens Rheumatoid Arthritis through the Activation of the Aryl Hydrocarbon Receptor and Interleukin-17 Pathways

**DOI:** 10.3390/antiox10060929

**Published:** 2021-06-07

**Authors:** Cintia Scucuglia Heluany, Paula Barbim Donate, Ayda Henriques Schneider, André Luis Fabris, Renan Augusto Gomes, Isadora Maria Villas-Boas, Denise Vilarinho Tambourgi, Tarcilia Aparecida da Silva, Gustavo Henrique Goulart Trossini, Giovanna Nalesso, Eduardo Lani Volpe Silveira, Fernando Queiroz Cunha, Sandra Helena Poliselli Farsky

**Affiliations:** 1 Department of Clinical and Toxicological Analyses, School of Pharmaceutical Sciences, University of São Paulo, São Paulo 05508-000, Brazil; cintiaheluany@usp.br (C.S.H.); afabris@usp.br (A.L.F.); eduardosilveira@usp.br (E.L.V.S.); 2 Department of Pharmacology; Ribeirão Preto Medical School, University of São Paulo, Ribeirão Preto 14049-900, Brazil; paulabarbim@usp.br (P.B.D.); ayda.hs@usp.br (A.H.S.); fdqcunha@fmrp.usp.br (F.Q.C.); 3 Department of Pharmacy, School of Pharmaceutical Sciences, University of São Paulo, São Paulo 05508-000, Brazil; renan2.gomes@usp.br (R.A.G.); trossini@usp.br (G.H.G.T.); 4 Immunochemistry Laboratory, Butantan Institute, São Paulo 05503-900, Brazil; isadora.boas@butantan.gov.br (I.M.V.-B.); denise.tambourgi@butantan.gov.br (D.V.T.); 5 Department of Oral Surgery and Pathology, School of Dentistry, Federal University of Minas Gerais, Belo Horizonte 31.270-901, Brazil; tarcilia@ufmg.br; 6 Department of Pre-Clinical Sciences, School of Veterinary Medicine, University of Surrey, Guildford GU2 7AL, UK; g.nalesso@surrey.ac.uk

**Keywords:** environmental pollutant, benzene metabolite, cigarette smoking, fibroblast-like synoviocytes, antigen-induced arthritis

## Abstract

Rheumatoid arthritis (RA) development is strongly associated with cigarette smoke exposure, which activates the aryl hydrocarbon receptor (AhR) as a trigger for Th17 inflammatory pathways. We previously demonstrated that the exposure to hydroquinone (HQ), one of the major compounds of cigarette tar, aggravates the arthritis symptomatology in rats. However, the mechanisms related to the HQ-related RA still remain elusive. Cell viability, cytokine secretion, and gene expression were measured in RA human fibroblast-like synoviocytes (RAHFLS) treated with HQ and stimulated or not with TNF-α. Antigen-induced arthritis (AIA) was also elicited in wild type (WT), AhR ^−/−^ or IL-17R ^−/−^ C57BL/6 mice upon daily exposure to nebulized HQ (25ppm) between days 15 to 21. At day 21, mice were challenged with mBSA and inflammatory parameters were assessed. The in vitro HQ treatment up-regulated TNFR1, TNFR2 expression, and increased ROS production. The co-treatment of HQ and TNF-α enhanced the IL-6 and IL-8 secretion. However, the pre-incubation of RAHFLS with an AhR antagonist inhibited the HQ-mediated cell proliferation and gene expression profile. About the in vivo approach, the HQ exposure worsened the AIA symptoms (edema, pain, cytokines secretion and NETs formation) in WT mice. These AIA effects were abolished in HQ-exposed AhR ^−/−^ and IL-17R ^−/−^ animals though. Our data demonstrated the harmful HQ influence over the onset of arthritis through the activation and proliferation of synoviocytes. The HQ-related RA severity was also associated with the activation of AhR and IL-17 pathways, highlighting how cigarette smoke compounds can contribute to the RA progression.

## 1. Introduction

Rheumatoid arthritis (RA) is a debilitating autoimmune disease characterized by synovial hyperplasia and severe chronic inflammation of the joints [1,2]. Enhanced proliferation of fibroblast-like synoviocytes (FLS) that constitute the synovial pannus is one of the RA hallmarks [3]. This pathogenic process is activated by pro-inflammatory cytokines that facilitate the release of reactive oxygen species (ROS), matrix metalloproteinases (MMPs) and growth factors [4]. The local inflammation is extended by the arrival of T and B lymphocytes, monocytes, dendritic cells and neutrophils that promote joint and bone destruction [4,5].

Indeed, the inflamed synovial cavity is composed by a complex chemical environment, of which tumor necrosis factor (TNF)-α and interleukin (IL)-17 exert pivotal actions in the RA pathophysiology. Within the sinovia, activated FLS, neutrophils, macrophages and lymphocytes primarily secrete TNF-α that binds to highly expressed receptors TNFR1 and TNFR2 [6]. This response also triggers the secretion of IL-1β, IL-23, IL-6, IL-8 and IL-17, resulting in a significant decrease in the frequency of regulatory T cells, favoring the establishment of a Th17 cell pattern [7]. The CD4+ T cell-secreted IL-17A can activate FLS, mature osteoclasts, and recruit neutrophils, macrophages and B cells to be activated [8,9]. In terms of the IL-17A relevance to this process, the treatment with an anti-IL-17A antibody has not been effective as the anti-TNF therapy is for RA patients [10]. However, models of experimental arthritis have shown that the deficiency of Th17 cells or other Th17 cytokines can reduce the RA severity or prevent the disease development [9,11].

The connection between genetic and environmental factors is known to influence the RA onset and evolution. Since the RA incidence has been highly reported in residents of industrialized countries, it has been suggested that the exposure to environmental pollutants may trigger and/or aggravate the disease [12]. Earlier reports have proposed that smoking is the major environmental risk factor involved in the RA pathogenesis [13,14]. In addition, smoking has been correlated with an increased and worsened RA incidence as well as to a diminished responsiveness to therapy [1,15].

Cigarette smoke is a complex aerosol system composed by roughly 8700 identified harmful substances, including nicotine, particulate matter, organic compounds, solvents, metals, gas substances and free radicals [16]. Many of them, such as radical species, impact the redox system, resulting in oxidative stress, inflammatory responses, carcinogenesis, and dysfunction of the immune cells [17,18,19]. In vitro studies with cigarette smoke condensate or isolated polycyclic aromatic hydrocarbons (PAHs) have pointed out the aryl hydrocarbon receptor (AhR) to participate in the RA aggravation in smokers [20,21,22]. Of note, smokers display an up-regulated level of AhR expression in their synovial membranes [23]. This receptor is a cytosolic transcription factor activated by ligands such as persistent organic pollutants, stimulating the expression of downstream genes. Among them, the cytochrome P450 (CYP) 1 family members are involved in the oxidation of endogenous hormones, drugs and xenobiotics [24]. Moreover, the AhR activation has been described as essential for the differentiation and activation of Th17 cells in the RA genesis [21,22,25].

Regarding the cigarette compounds, the hydroquinone (HQ) has been particularly associated with oxidative stress in the particle matter phase and is detected in about 100 μg/cigarette [16]. During the biotransformation of benzene, the HQ is also generated and can cause myelotoxicity and immunotoxicity in the lungs, liver and bone marrow [26]. Our research group has pointed out the HQ toxic properties to the immune system, especially those that cause the impairment of innate and acquired immune responses [27,28,29]. Another harmful effect of the HQ exposure has being the age-related macular degeneration, a disease with high incidence in smokers [30,31].

Also, we recently demonstrated that the experimental model of collagen-induced arthritis (CIA) in rats can be significantly intensified upon the HQ exposure. The synovia of these HQ-exposed CIA rats presented elevated levels of IL-6, higher inflammation, pannus formation and increased influx of immune AhR+ and IL-17+ cells [32,33]. Herein, we aimed to investigate the involvement of AhR and IL-17 in the mechanism of action in the worsened HQ-related arthritis and evaluate the outcome of RA human fibroblast-like synoviocytes (RAHFLS) exposed to HQ, associated or not with the activation of the TNF-α pathway. Our data clearly show the HQ influence over the synoviocyte activation, cytokine secretion and oxidative stress, and confirm the involvement of AhR and IL-17R on the exacerbated experimental arthritis in vivo.

## 2. Materials and Methods

### 2.1. Chemicals and Reagents

The following materials were used: complete Freund’s adjuvant (CFA; Sigma-Aldrich, St. Louis, MO, USA; #F5881); incomplete Freund´s adjuvant (IFA; Sigma-Aldrich, St. Louis, MO, USA); hydroquinone (Hydroquinone 99%—Sigma-Aldrich, St. Louis, MO, USA); TNF-α (#DY410), IL-6 (#DY406) and IL-17 (#DY421) mouse ELISA kits (R&D Systems, Minneapolis, MN, USA); IL-6 (#33-7068-68) and IL-8 (88-8086-88) human ELISA kits (Thermo Fisher Scientific, Waltham, MA, USA); Dulbecco’s modified Eagle’s medium F12 (DMEM F12, Thermo Fisher, Waltham, MA, USA); fetal bovine serum (FBS; GIBCO Invitrogen, Carlsbad, CA, USA), penicillin and streptomycin (Invitrogen, Carlsbad, CA, USA); human TNF-α (Sigma-Aldrich, St. Louis, MO, USA); bovine serum albumin (BSA, Sigma-Aldrich, St. Louis, MO, USA); dimethyl sulfoxide (DMSO, Sigma-Aldrich, St. Louis, MO, USA); phorbol 12-myristate 13-acetate (PMA, Sigma-Aldrich, St. Louis, MO, USA); Cytofix/Perm kit (BD Biosciences, San Jose, CA, USA); 3-(4,5-dimethylthylthiazol-2-yl)-2,5 diphenyltetrazolium bromide CM-H2DCFDA (Invitrogen, Carlsbad, CA, USA); PE-conjugated anti-CD90 antibody (clone eBio5E10; eBioscience, San Diego, CA, USA); anti-AhR antibody (RPT1; Abcam, Cambridge, UK); goat anti-mouse IgG Alexa Fluor 488 antibody (Life Technologies, Carlsbad, CA, USA); Vectashield mounting solution containing 40, 6-diamidino-2-phenylindole (DAPI) (Vector Laboratories, Burlingame, CA, USA); anti-TNFR1 antibody (Rabbit polyclonal antibody to TNF Receptor I, Abcam, Cambridge, UK), anti-TNFR2 antibody (Rabbit polyclonal antibody to TNF Receptor II, Abcam, Cambridge, UK); rabbit anti-citrullinated H3 primary antibody; goat anti-rabbit IgG Alexa Fluor 488 antibody (Invitrogen, Carlsbad, CA, USA); AhR antagonist α-naphthoflavone (αNF, Sigma-Aldrich, St. Louis, MO, USA); methylated bovine serum albumin (mBSA; Sigma-Aldrich, St. Louis, MO, USA); RNeasy Mini Kit (Qiagen, Hilden, Germany); High Capacity Kit (Life Technologies, Carlsbad, CA, USA); Taqman Gene Expression Master Mix (Thermo Fisher Scientific, Waltham, MA, USA).

### 2.2. Cell Culture and Phenotypical Characterization

Primary human fibroblast-like synoviocytes from RA patients (RAHFLS) were purchased from Articular Engineering (Northbrook, IL, USA), and cultured in DMEM F12 culture medium supplemented with 10% FBS, 100 U/mL penicillin, and 100 μg/mL streptomycin at 37 °C in a 5% CO_2_ incubator. Three to nine cell passages were utilized in experiments of this study.

To confirm that RAHFLS could maintain the arthritic phenotype along the culture passages, even upon the HQ exposure, we verified their ability to express CD90 (Thy-1), a glycosylphosphatidylinositol (GPI)-linked cell surface glycoprotein that indicates enhanced proliferantion [34] and is highly expressed in the synovia of RA patients [35], through FACS. For this, 1 × 10^4^ cells were seeded per well in 24-well plates and treated with HQ (1 or 10 µM) and/or with TNF-α (2 ng/mL) for 24 h. Thereafter, cells were harvested, fixed and stained with a PE-conjugated anti-CD90 antibody (diluted 1:100 in PBS + 0.1% BSA + 0.01% sodium azide) during 1 h at 37 °C. Then, 10,000 events were acquired in a Accuri C6 cytometer (BD Biosciences, San Jose, CA, USA). The flow cytometry analysis was carried out with the FlowJo software (Tree Star Inc, Ashland, OR, USA). Results are presented as arbitrary units of fluorescence.

### 2.3. Animals

Adult male wild-type (WT) C57BL/6, AhR genetic-deficient (AhR^−/−^) and IL-17 receptor genetic-deficient (IL-17R^−/−^) mice were bred in a specific pathogen-free animal facility at the School of Medicine of Ribeirão Preto (University of São Paulo, Brazil). Animals were maintained in sterile, isolated, ventilated cages with controlled temperature, light conditions and were supplemented with food and water *ad libitum*. All the genetic-deficient mice (AhR^−/−^ and IL-17R^−/−^) displayed overall good health conditions and optimal breeding. All procedures were performed according to the guidelines of the Brazilian Society of Science of Laboratory animals for the proper care and use of experimental animals. Experimental procedures were approved by the Ethics Committee on Animal Use from the University of São Paulo (protocol numbers 563; 048/2012). AhR^−/−^ mice on the C57BL/6 background were provided by Dr Frank Gonzalez (National Cancer Institute, National Institute of Health, Bethesda, USA) and IL-17R^−/−^ mice on the C57BL/6 background were provided by Professor Jay Kolls (University of Pittsburgh, School of Medicine, USA). All WT and KO animals have the same C57BL/6 genetic background but are not considered true littermates.

### 2.4. In Vivo Hydroquinone (HQ) Exposure

Mice were daily exposed to a HQ solution at 25 ppm (1.5 mg/60 mL) through an ultrasonic nebulizer (NS^®^ São Paulo, SP, Brazil) for 1 h during 7 consecutive days after the second immunization (between days 15 to 21), as previously described [32,33]. As control, animals were exposed to HQ vehicle solution (5% ethanol in saline) during the same period.

### 2.5. Antigen-Induced Arthritis (AIA)

Animals were anesthetized with 2% isoflurane before immunization and challenge. On day 0, mice were subcutaneously (s.c.) injected with 500 µg of mBSA in prepared 200 µL of an emulsion containing equal volumes of saline and Complete Freund´s Adjuvant (CFA). On days 7 and 14, mice were boosted via s.c. with the same protein adjuvanted with the Incomplete Freund´s Adjuvant (IFA). A week later, mice were challenged with an intra-articular (i.a.) mBSA injection (30 µg diluted in 10 µL of PBS) into the right knee joint.

### 2.6. Assessment of Articular Hyperalgesia and Edema

Arthritic symptoms, such as articular hyperalgesia of the joints and edema, were determined 6 h after the mBSA challenge as previously described [36]. Briefly, animals were placed in acrylic cages with wire grid floors in a silent room for environmental adaptation 30 min before the tests. To measure hyperalgesia, the animals need at least one of the paws without pain to support their weight in the cages and the measurements were performed only when mice did not present exploratory movements. The electronic pressure-meter utilized in this assay consisted of a hand-held force transducer fitted with a polypropylene tip (4.15 mm^2^) (IITC Life Science Instruments, Woodland Hills, CA, USA). An increasing perpendicular force was applied to the central area of the plantar surface of the hind paw to induce flexion of the femur-tibial joint followed by the paw withdrawal. Thus, the pressure of the applied force was recorded through the pressure meter when the paw was withdrawn. Whereas the mechanical threshold was expressed in grams (g), the hyperalgesia was equated to the reduction of this threshold.

The edema formation was determined in the femur-tibial joint area using a caliper before and 6 h after the mBSA challenge. The edema unit was expressed in millimeters through the quantification of paw volumes.

### 2.7. Synovial Fluid Collection and Measurement of Cell Influx

The synovial fluid was obtained 6 h after the mBSA challenge upon the mouse euthanasia, through 2 injections of 10 µL of PBS in the femur-tibial articular cavities. Cells were diluted in Turk´s solution and their total number was determined through cell counting on a Neubauer chamber. Leukocytes subpopulations were determined by performing a cytospin method followed by a differential cell counting method using a commercial kit (Panotico; Renylab, Barbacena, MG, Brazil), based on Romanowsky´s staining method [37]. Results were expressed as the number of cells per joint.

### 2.8. Histopathological and Immunofluorescence Analyses

Murine femur-tibial joints (4 animals per group) were collected 24 h after the mBSA challenge upon the animal euthanasia. Then, joint tissues were fixed in 10% formalin, decalcified in 10% EDTA solution (pH 7.4) during 21 days and histologically processed and analyzed. Tissues were embedded in paraffin and 5 μm sagittal sections were obtained, stained in hematoxylin & eosin (H&E) and analyzed. Three sections/knee joint were microscopically examined by a single pathologist (T.A.S.) and scored in a blind manner for different parameters, as follows: severity of synovial hyperplasia and/or discontinuity (0–4); and intensity and extension of influx of inflammatory cells (0–5). The grades were summed to obtain an arthritis index (ranging from 0 to 9), with the results expressed as the mean histopathological score, according to Willians et al. 2007 [38]. Images were acquired through 4× magnification objective lenses, using an Axioskop 40 microscope (CarlZeiss, Oberkochen, Germany) adapted to a digital camera (Canon PowerShot A620, Tokyo, Japan).

The formation of neutrophil extracellular traps (NETs) was indicated through indirect immunofluorescence. Tissue slices were processed and stained overnight with an anti-citrullinated H3 primary antibody (diluted 1:1000 in PBS + 0.1% BSA + 0.01% Triton-X-100) at 4 °C. After 3 washing steps with PBS, the slides were stained with a goat anti-rabbit IgG Alexa Fluor 488 secondary antibody (diluted 1:2000 in PBS + 0.1% BSA+ 0.01% Triton-X-100), for 2 h at room temperature. Slides were mounted with Vectashield containing DAPI and analyzed on a confocal microscope (TCS SP5, Leica Microsystems, Wetzlar, Germany). In order to obtain a representative image of the real AIA effect on the knee joints, we initially scanned the total area of the slides, always using the same magnification (images were acquired through 40× magnification objective lenses with 2× zoom). Afterwards, we selected 4 different tissue areas to analyze. The ImageJ software (National Institutes of Health, Bethesda, MD, USA) was used to quantify the fluorescence intensity in the areas of interest for each slide. At least, 4 fields per slide (totaling 16 images per experimental group) were considered for the final analysis. Then, the mean intensity values were extracted of data derived from 4 sections of each knee joint and used for the statistical analysis.

### 2.9. Cell Viability

Cell viability was measured through the 3-(4,5-dimethylthylthiazol-2-yl)-2,5 diphenyltetrazolium bromide (MTT) method. Briefly, 1 × 10^4^ RAHFLS were seeded per well in 96-well plates and cultured with DMEM F12 culture medium supplemented with 10% FBS for 24 h. Thereafter, cells were washed with PBS and incubated with DMEM F12 culture medium supplemented with 0.1% of BSA for 24 h. After another washing step, cells were incubated with DMEM F12 culture medium supplemented with 2% FBS or HQ (1 or 10 µM) for 24 h. Then, plates were washed and incubated with MTT solution (0.5 mg/mL–100 µL/well) for 3 h in the dark at 37 °C. Subsequently, the plates were washed with PBS and 200 µL of DMSO were added to each well. Plates were shaken for 5 min and the optical density reading was obtained through spectrophotometry at 570 nm. DMEM F12 culture medium supplemented with 10% FBS was used as positive control, and the BSA addition synchronized all cells in the G_0_ phase.

### 2.10. Cytokine Quantification

Cytokines related to the RA onset were quantified upon in vitro and in vivo approaches in this study. Initially, 1 × 10^4^ RAHFLS were seeded per well in 48-well plates and treated with HQ (1 or 10 µM) for 4 h, followed by the incubation with or without TNF-α (2 ng/mL) for 20 h. The inverse protocol was also attempted, in which cells were pre-treated with TNF-α for 4 h, followed or not by the HQ treatment (1 or 10 µM) for 20 h. The supernatants were collected 24 h later for the quantification of human IL-6 and IL-8 through ELISA according to the manufacturer’s instructions (Thermo Fisher Scientific, Waltham, MA, USA).

The same type of assay was used to measure the cytokine concentrations in homogenates of murine knee joints. To prepare the tissue homogenates, the murine femur-tibial joints were collected, frozen in liquid nitrogen, macerated in a metal apparatus with the aid of a hammer, ressuspended in 500 µL of PBS containing protease inhibitor (25×) (Roche), and centrifuged for 10 min, 4 °C, at 1500× *g*. After that, the supernatants were collected and stored at −70 °C until used to quantify TNF-α, IL-6 and IL-17 concentrations according to the manufacturer’s instructions (R&D Systems, Minneapolis, MN, USA). All results are expressed as picograms (pg) of cytokine produced per supernatant per milliliter (mL) for human and mouse cytokines.

### 2.11. Protein Expression and Cell Frequency Measurements

To evaluate the effects of the HQ exposure on the expression of TNFR1 and TNFR2, indirect immunofluorescence assays were performed. For the TNF receptors, 1 × 10^4^ RAHFLS were plated in culture slides (8 Chamber Polystyrene Vessel Tissue Culture Treated Glass Slide, BD Falcon, San Jose, CA, USA) and treated with HQ (1 or 10 µM) in the presence or not of TNF-α (2 ng/mL) for 24 h. Then, cells were washed with PBS, fixed in cold methanol for 20 min at −20 °C, followed by the cell permeabilization with 0.01% Triton-X-100 for 20 min. After rinsing slides 3 times with PBS, cells were treated with 5% FBS diluted in PBS to reduce nonspecific background staining for 1 h. Slides were rinsed with PBS again and cells were incubated with anti-TNFR1 or anti-TNFR2 primary antibodies, (diluted 1:100 in PBS + 0.1% BSA + 0.01% sodium azide), for 1 h at 37 °C, according to Xiao et al., 2017 [39]. After 3 washing steps, cells were stained with goat anti-rabbit IgG Alexa Fluor 488 secondary antibody, diluted 1:200 in PBS + 0.1% BSA + 0.01% sodium azide, for 1 h at room temperature. Slides were mounted with Vectashield containing DAPI and analyzed on a fluorescent imaging microscope (Imager.A2 Axio, Carl Zeiss, Oberkochen, Germany). Images were acquired with 100× magnification objective lenses and analyzed with the ImageJ software (National Institutes of Health, Bethesda, MD, USA). The ImageJ software was used to quantify the fluorescence intensity in the areas of interest for each culture slide. At least, 4 fields per well (totaling 12 images per experimental group) were considered for the final analysis. Then, the mean intensity values were extracted of data derived from 4 regions of each slide and used for the statistical analysis.

### 2.12. In Vitro Detection of Reactive Oxygen Species

The intracellular accumulation of reactive oxygen species (ROS) was monitored in cell cultures using the fluorescent probe CM-H2DCFDA. Briefly, 1 × 10^4^ RAHFLS were seeded per well in 24-well plates and incubated with HQ (1 or 10 µM) in the presence or not of TNF-α (2 ng/mL) for 24 h. Then, 10 μM CM-H2DCFDA were added to the cells, whose incubation was performed at 37 °C for 30 min in the dark. After a washing step, cells were ressuspended in PBS and 10,000 events were acquired in an Accuri C6 cytometer (BD Biosciences, San Jose, CA, USA). Phorbol 12-myristate 13-acetate (PMA, 50 nM) was used as positive control. Results were presented as arbitrary units of fluorescence.

### 2.13. Influence of AhR Antagonist in RAHFLS Proliferation, TNFR Expression and ROS Generation

To investigate whether the HQ-derived cellular alterations were triggered through AhR activity, 1 × 10^4^ RAHFLS were plated per well in a 24-well plate and pre-treated with the AhR antagonist α-naphthoflavone (αNF, 100 µM) for 30 min. Then, cells were treated with HQ (1 or 10 µM) for 24 h, followed by the staining with a PE-conjugated anti-CD90 antibody (diluted 1:100) as previously described. AhR^+^ cells were quantified through flow cytometry as described above. The same AhR antagonist had its effect evaluated over the expression profile of TNF receptors and ROS generation. Regarding the TNFR expression, 1 × 10^4^ RAHFLS were plated per well in an 8-chamber culture slides and pre-treated with αNF (100 µM) for 30 min. Then, cells were treated with HQ (1 or 10 µM) for 24 h. Afterwards, cells were processed, stained with anti-TNFR1 or anti-TNFR2 antibodies and analyzed on a fluorescent imaging microscope as previously described. About the ROS generation, 1 × 10^4^ RAHFLS were seeded per well in 24-well plates and pre-treated with αNF (100 µM) for 4 h. Then, cells were incubated with HQ (1 or 10 µM) for 20 h, followed by the 10 μM CM-H2DCFDA loading. The intracellular ROS accumulation was further analyzed through flow cytometry as described above.

### 2.14. Quantification of mRNA Expression

For the gene expression analysis, 1 × 10^5^ RAHFLS were plated per well in a 6-well and treated with HQ (1 or 10 µM) for 30 min. Total RNA (50 ng) was extracted using RNeasy Mini Kit following manufacturer´s instructions (Qiagen, Hilden, Germany), and reverse-transcribed to cDNA with the High Capacity Kit. Real time PCR assays were performed using Taqman Gene Expression Master Mix. Taqman gene assay numbers: *Cyp1a1* (Hs01054797_g1) and *Gapdh* (Hs04420632_g1). All data were normalized to Gapdh values as an internal control. All experiments were performed into a Viia7 Real-time PCR system (Thermo Fisher Scientific, Waltham, MA, USA), and the comparative threshold cycle method was used to determine the relative expression levels.

### 2.15. Statistical Analysis

The data sets were subjected to normality tests and analyzed by one-way analysis of variance (ANOVA) followed by Tukey’s post hoc test. The level of significance adopted was 95% (*p* < 0.05). All data are represented as mean ± standard error of the mean (S.E.M.). All calculations were performed with GraphPad Prism version 7.0 (GraphPad Software, San Diego, CA, USA).

## 3. Results

### 3.1. In Vitro HQ Exposure Induces RAHFLS Activation

**Figure 1:**
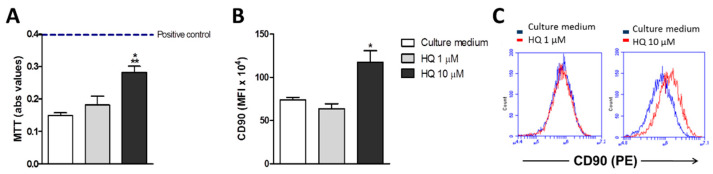
In vitro HQ exposure enhances the viability of RAHFLS. RAHFLS (1 × 10^4^ cells) were incubated with culture medium or with HQ (1 or 10 µM) for 24 h, and had their viability and ability to proliferate determined through the MTT method (**A**) and the flow cytometry method based on the staining with an anti-CD90 antibody (**B**,**C**). Data represent mean ± SEM from three independent experiments and were analyzed by one-way ANOVA (**A**): ** *p* < 0.01 vs. culture medium; * *p* < 0.05 vs. HQ 1 µM; (**B**): * *p* < 0.05 vs. culture medium and vs. HQ 1 µM. (**A**): positive control: DMEM F12 culture medium supplemented with 10% FBS.

To investigate the HQ effects over primary FLS from RA patients (RAHFLS), cells were incubated with different HQ concentrations (1–100 μM) for 24 h. Whereas the HQ treatments with 1 μM and 10 μM could maintain the viability of synovial cells, the highest HQ concentration (100 μM) could not (Supplementary Materials Figure S1A–D). Thus, the HQ treatment with 100 μM was excluded from further analyses. The RAHFLS showed an increased activation after 24-h incubation with 10 μM of HQ in comparison to cells cultured with the lowest HQ concentration or culture medium (Figure 1A).

During the onset of RA, activated synoviocytes proliferate in response to inflammatory mediators, such as TNF-α [4] and CD90 is a marker that indicates enhanced proliferation [34]. In fact, we observed around 92% of CD90+ cells after the CD90 staining. The HQ treatment with 10 μM showed not only a higher CD90 staining of synoviocytes (Figure 1B), but also that the majority of cells were CD90+, in comparison to the other tested conditions, suggesting that the HQ exposure may augment the proliferation capacity of synoviocytes (Figure 1C).

### 3.2. In Vitro HQ Exposure Potentiates the Cytokine Secretion and Enhances TNFR1 and TNFR2 Expression in TNF-α-Treated RAHFLS

**Figure 2:**
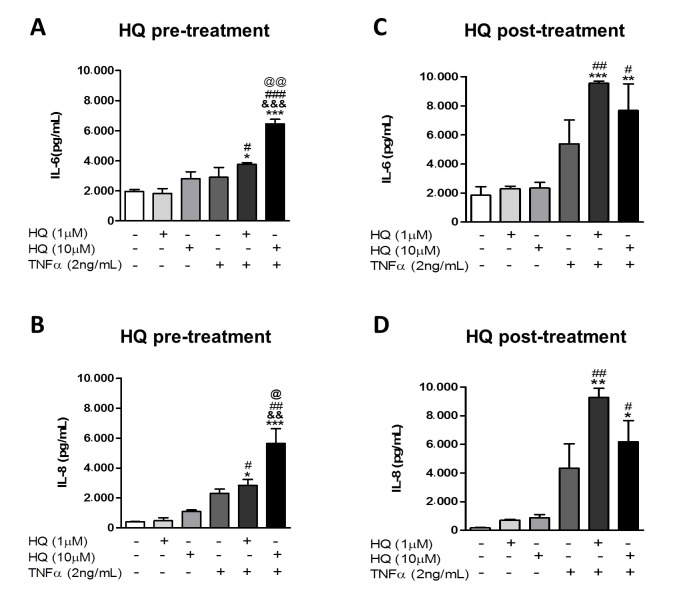
In vitro HQ exposure potentiates the production of pro-inflammatory cytokine by RAHFLS when combined with TNF-α treatment. RAHFLS (1 × 10^4^ cells) were pre- (**A**,**B**) or post-incubated (**C**,**D**) with culture medium or with HQ (1 or 10 µM) for 4 h, followed by the incubation with or without TNF-α (2 ng/mL) for 20 h. Supernatants were harvested and had the levels of interleukin (IL)-6 and -8 quantified through ELISA. Data represent mean ± SEM from three independent experiments and were analyzed by one-way ANOVA. * *p* < 0.05, ** *p* < 0.01 and *** *p* < 0.001 vs. culture medium; ^#^  *p* < 0.05, ^##^ *p* < 0.01 and ^###^ *p* < 0.001 vs. respective groups without TNF-α; ^&&^ *p* < 0.01 and ^&&&^ *p* < 0.001 vs. TNF-α; ^@^ *p* < 0.05 and ^@@^ *p* < 0.01 vs. HQ 1 µM + TNF-α.

The TNF-α effects in the RA progression are mediated by its binding to specific receptors (TNFR1 and TNFR2) expressed in synoviocytes, triggering the secretion of inflammatory cytokines, such as IL-6 and IL-8 [6]. Hence, we investigated the HQ effect over the cytokine secretion in RAHFLS cultures. Our data showed that both pre- and post-HQ treatments could enhance the secretion of IL-6 and IL-8, but only in the presence of TNF-α (Figure 2A–D). Therefore, the HQ exposure clearly strengthens the ability of TNF-α-treated RAHFLS to secrete inflammatory cytokines, which may also be a potential mechanism to worsen RA in patients. Despite the importance of other cytokines in the RA pathogenesis, such as IL-17 and IL-10, we have not quantified their production in this study as they are not produced by synoviocytes [40].

**Figure 3:**
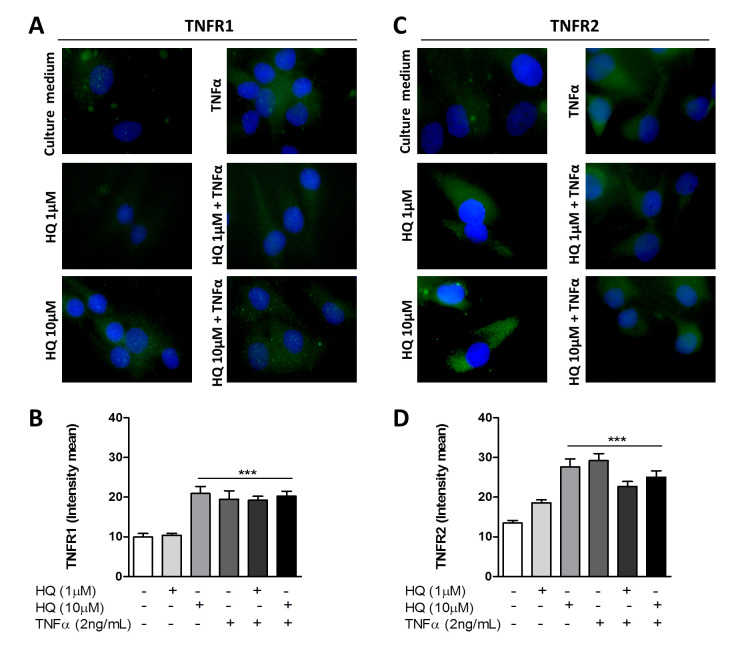
In vitro HQ treatment induces TNFR1 and TNFR2 expression in RAHFLS. RAHFLS (1 × 10^4^ cells) were incubated with culture medium, or TNF-α (2 ng/mL) or HQ (1 or 10 µM) in presence or absence of TNF-α (2 ng/mL) for 24 h. Then, the expression of TNFR1 (**A**) and TNFR2 (**C**) were quantified through an indirect immunofluorescence assay and the mean intensity of immunoreactive areas were quantified (**B**,**D**). DAPI—positive staining for nuclei. Original magnification—100×. Data represent mean ± SEM from three independent experiments and were analyzed by one-way ANOVA. *** *p* < 0.001 vs. culture medium or HQ 1 µM.

Furthermore, it remained elusive whether the HQ effect over RAHFLS was restricted to cytokine secretion or if it could also affect earlier stages of that process, such as the expression of TNF-α receptors. Thus, we sought to determine whether the HQ exposure could up-regulate the expression of TNFR1 and TNFR2 in TNF-α-treated RAHFLS. Both HQ (10 μM) and TNF (2 ng/mL) induced a significant up-regulation of TNFR1 and TNFR2 in RAHFLS cultures. However, the combined treatment of TNF-α (2 ng/mL) and HQ (1 μM and 10 μM) for 24 h did not enhance the expression of TNFR1 and TNFR2 (Figure 3A–D). Thus, our data suggest that the presence of HQ in the synovial environment can promote the expression of TNF-α receptors in RAHFLS. Once activated by TNF-α, the triggered intracellular signaling can potentially stimulate a particular profile of cytokine secretion associated to the aggravation of the RA symptomatology.

The transcription factor NF-κB is responsible for the transcriptional activity of pro-inflammatory mediators. For this, an important upstream step is the nuclear translocation of the NF-κB p65 unit and further NF-Κb activation [41]. As expected, the TNF-α treatment promoted the upregulation of NF-κB p65 in RAHFLS. On the other hand, distinct concentrations of HQ were not able to augment this readout in the same cell culutre, suggesting that HQ could activate other pro-inflammatory pathways in RAHFLS (Supplementary Materials Figure S2).

### 3.3. In Vitro HQ Exposure Triggers the ROS Generation in RAHFLS

**Figure 4:**
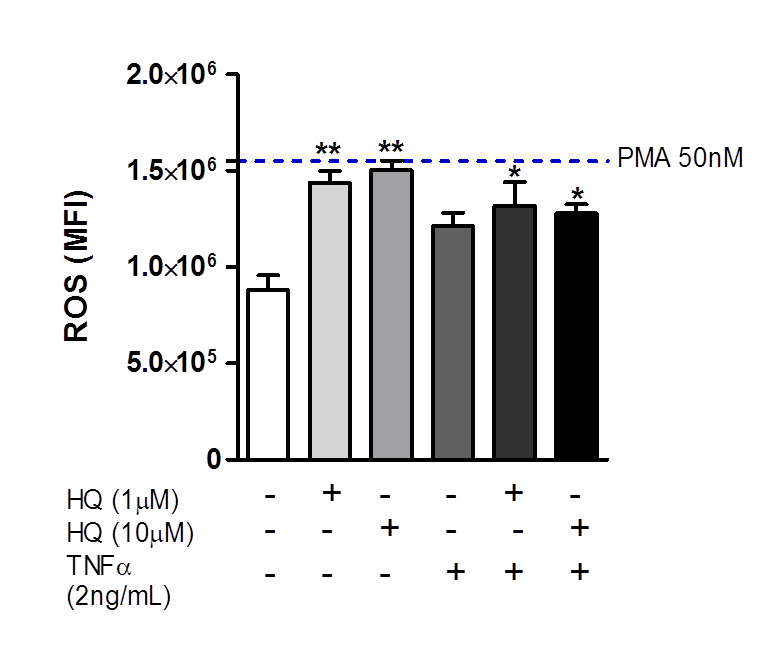
In vitro HQ treatment enhances the ROS generation by RAHFLS. RAHFLS (1 × 10^4^ cells) were incubated with culture medium or HQ (1 or 10 µM) with or without TNF-α (2 ng/mL) for 24 h. ROS was quantified in synoviocytes using DCFH-DA assay. Data represent mean ± SEM from three independent experiments and were analyzed by one-way ANOVA. * *p* < 0.05 and ** *p* < 0.01 vs. culture medium.

The induction of oxidative stress in synoviocytes has been strongly associated with the RA severity in patients [42]. Since HQ is known to facilitate the ROS generation in several cell subsets [32,33,43,44], we wondered whether the exposure to this toxic compound could impact the ROS generation in FLS cultures. Thus, we demonstrated that the incubation with 1 or 10 µM of HQ elicited a significant increase in the ROS production, similar to those presented by PMA-treated RAHFLS (positive control), relative to untreated synoviocytes (Figure 4).

### 3.4. AhR Activity Is Involved in the HQ-Induced RAHFLS Proliferation and TNFRs Expression

**Figure 5:**
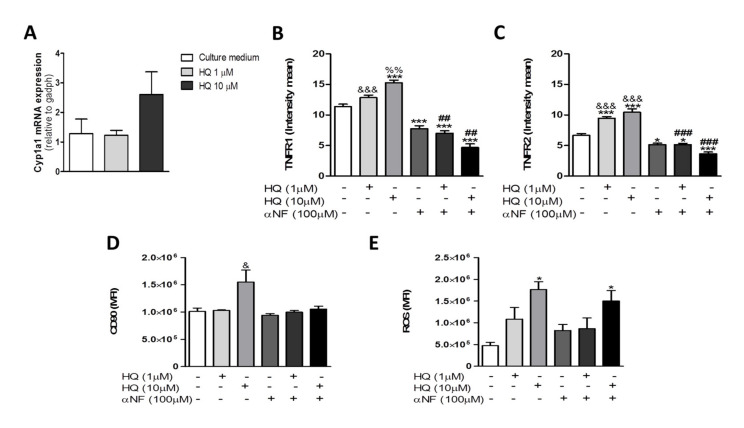
In vitro AhR antagonist treatment prevents cell proliferation and TNFRs expression evoked by HQ exposure. RAHFLS (1 × 10^4^ cells) were treated with HQ (1 or 10 µM) with or without the AhR antagonist α-naphthoflavone (αNF, 100 µM). After 30 min of treatments, the Cyp1a1 mRNA expression was quantified by RT-PCR (**A**). After 24 h of treatments, the TNRF1 and TNFR2 expression were quantified by indirect immunofluorescence assay in the synovial cells (**B**,**C**) and the synovial proliferation was quantified through flow cytometry (**D**). The ROS generation was quantified using DCFH-DA assay (**E**). Data represent mean ± SEM of three independent experiments in RAHFLS. Data were analyzed by one-way ANOVA. * *p* < 0.05 and *** *p* < 0.001 vs. culture medium; ^&^ *p* < 0.05 and ^&&&^ *p* < 0.001 vs. αNF; ^%%^ *p* < 0.01 vs. HQ 1 µM; ^@@^ *p* < 0.01 vs. HQ 10 µM + αNF; ^##^ *p* < 0.01 and ^###^ *p* < 0.001 vs. respective groups without αNF.

We have previously shown that the in vivo HQ exposure significantly increased the frequency of AhR^+^ cells in the synovial membrane of knee joints from CIA-rats during the late phase of the experimental RA model disease in comparison to their counterparts [32,33]. Similar effects were also detected in HQ-exposed CIA-rats during both the sensitization and the final RA phases (Supplementary Materials Figure S3A,B). Thus, we decided to investigate the HQ influence on the regulation of the AhR activity in human synoviocytes. Since the AhR activation has been associated with the transcription of metabolic enzymes belonging to the CYP1 family [45], we quantified the Cyp1a1 transcripts in HQ-exposed RAHFLS. Despite the lack of a significant difference among groups, RAHFLS treated with 10 μM of HQ tended to up-regulate the Cyp1a1 mRNA expression in comparison to cells exposed to HQ 1 μM and untreated cells (Figure 5A).

To ensure the AhR relevance in the RA-related processes, we treated the RAHFLS with alpha-naphthoflavone (αNF, 100 μM), a pharmacological AhR antagonist, prior to the HQ treatment and evaluated the effects of this co-stimulation on the synovial proliferation, ROS generation and TNFRs expression. Of note, the AhR antagonist pre-treatment hindered the increased expression of TNFR1 (Figure 5B and Supplementary Materials Figure S4A) and TNFR2 (Figure 5C and Supplementary Materials Figure S4B) and the synovial proliferation (Figure 5D) elicited by the HQ exposure with 10 μM. However, the αNF treatment did not alter the enhanced ROS levels triggered by the 10 μM of HQ stimulation in RAHFLS cultures (Figure 5E).

In order to confirm the possibility of interaction between HQ and AhR, we used an in-silico approach to investigate the potential HQ-AhR interaction mapping. For this, the AhR surface was scanned through FTMap for putative binding site recognition and the most representative cluster of molecular probes were located close to the A’α helix and A, H and I β-sheets (Supplementary Materials Figure S5A). Among the 22 probes in the cluster, three of them (a phenol and two benzaldehydes) had the same structural features as the investigated ligand (Supplementary Materials Figure S5B), an aromatic ring and hydrogen bond acceptors (HBAs) as substituent groups. These three probes were interacting with different residues, but the R236 in the Hβ was the only shared (polar or non-polar) contact among them. After superimposing their predicted binding modes, the HBAs were located at opposite sides of the benzene ring (Supplementary Materials Figure S5C), as on the hydroquinone structure. These findings converged with the predicted binding mode generated by molecular docking (Supplementary Materials Figure S5D). The HQ was not only interacting with the R236 residue, but also had hydrogen bonds connecting with E211 and Q234 and Van der Waals contacts with G235 and I262. Hence, this network of strong polar interactions in a small cavity suggested by this consensus analysis supports a plausible binding mode for HQ. These results corroborate with both of our in vitro and in vivo findings.

### 3.5. AhR and IL-17 Are Involved in the AIA Worsening upon an In Vivo HQ Exposure

**Figure 6:**
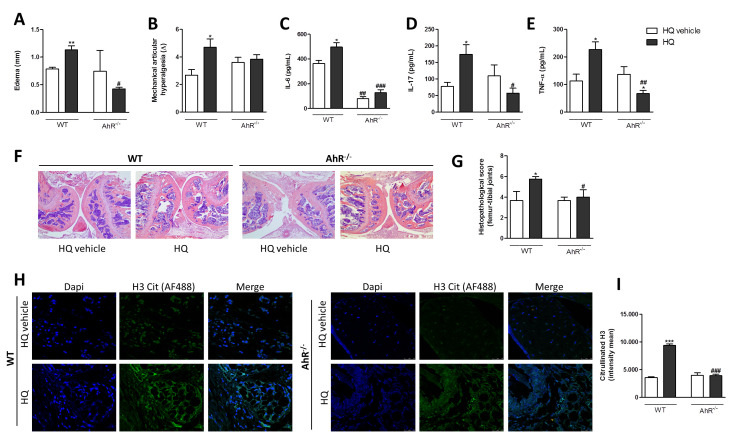
AhR is involved in the AIA aggravation after the in vivo HQ exposure. C57BL/6 WT and AhR^−/−^ mice were exposed to HQ or vehicle, 1 h/day between days 15–21 after the first immunization. On the 21th day after the first immunization, animals were challenged with mBSA. Six hours later, mice were euthanized and edema (**A**), mechanical articular hyperalgesia (**B**), quantification of cytokines IL-6 (**C**) and IL-17 (**D**), TNF-α (**E**), histopathological analyses (**F**,**G**) and indirect immunofluorescence analyses of NET formation by assessing the quantification of H3 citrullination (**H**,**I**). (**F**): original magnification—4×. (**H**): original magnification—40×. Data represent mean ± SEM of four animals in each group and were analyzed by one-way ANOVA. * *p* < 0.05, ** *p* < 0.01 and *** *p* < 0.01 vs. HQ vehicle; ^#^ *p* < 0.05, ^##^ *p* < 0.01 and ^###^ *p* < 0.001 vs. respective group in WT mice.

We have previously demonstrated that the HQ exposure worsens CIA in rats with increased infiltrate of AhR^+^ and IL-17^+^ cells in the synovia [32,33]. To investigate the HQ mechanism of action in vivo, we induced AIA in mice deficient in AhR (AhR^−/−^) or IL-17R (IL-17R^−/−^). The exposure to nebulized HQ in the last 7 days of the disease enhanced edema (Figure 6A), hypernociception (Figure 6B), levels of IL-6, IL-17 and TNF-α (Figure 6C–E) and the mean histopathological score (characterized by synovial hyperplasia and influx of inflammatory cells) in the femur-tibial joints of AIA-WT animals (Figure 6F,G). Regarding the increased influx of inflammatory cells into the articular cavity, mainly characterized by neutrophils, it was observed that the HQ exposure also triggered a higher incidence of H3 citrullination, which is an indicative of NET formation in the femur-tibial joints of WT mice (Figure 6H,I). However, none of these HQ-related AIA effects were observed in AhR^−/−^ mice (Figure 6A–I).

**Figure 7:**
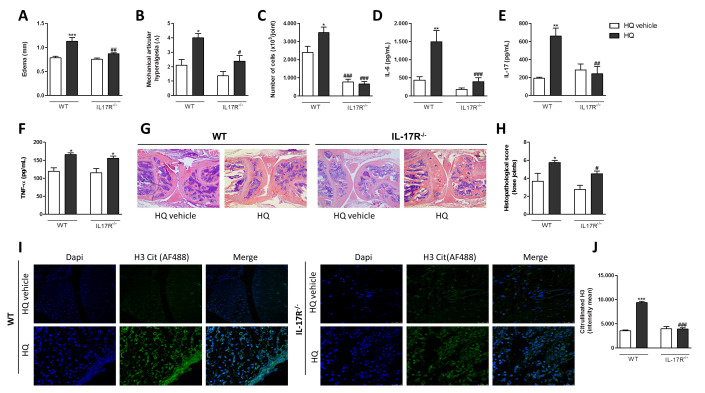
IL-17 is involved in the AIA aggravation triggered by the in vivo HQ exposure. C57BL/6 WT and IL-17R^−/−^ mice were exposed to HQ or vehicle, 1 h/day between days 15–21 after the first immunization. On the 21th day after the first immunization, animals were challenged with mBSA. Six hours later, mice were euthanized and edema (**A**), mechanical articular hyperalgesia (**B**), influx of cells into the joints (**C**), quantification of cytokines IL-6 (**D**) and IL-17 (**E**) and TNF-α (**F**), histopathological analyses (**G**,**H**) and indirect immunofluorescence analyses of NET formation by assessing the quantification of H3 citrullination (**I**,**J**) were assessed. (**G**): original magnification—4×. (**I**): original magnification—40×. Data represent mean ± SEM of four animals in each group and were analyzed by one-way ANOVA. * *p* < 0.05, ** *p* < 0.01 and *** *p* < 0.001 vs. HQ vehicle; ^#^ *p* < 0.05, ^##^ *p* < 0.01 and ^###^ *p* < 0.001 vs. respective group in WT mice.

Regarding the role of IL-17R in this disease pathophysiology, this receptor seems to have a direct participation on its process. AIA-IL-17R^−/−^ mice exposed to HQ displayed neither augmented edema (Figure 7A), hypernociception (Figure 7B), nor neutrophil influx to the synovia cavity (Figure 7C) as observed in AIA-WT mice. The IL-6 and IL-17 levels were also diminished in the synovia of AIA-IL-17R^−/−^ mice in comparison to AIA-WT mice (Figure 7D,E). The exception was only the TNF-α level that was similar between AIA-WT and -IL-17R^−/−^ mice exposed to HQ (Figure 7F). Moreover, synovial hyperplasia, influx of inflammatory cells in the femur-tibial joints and NET formation on the knee joints were strongly reduced in AIA-IL-17R^−/−^ mice relative to AIA-WT mice (Figure 7 G–J).

## 4. Discussion

The connection between the exposure to environmental pollutants and the adverse effects in humans undoubtedly reflects the rise of severe public health problems, especially to chronic diseases. RA is a debilitating chronic disorder, of which the genesis has been strongly linked to pollutant exposure [12,46]. Our group has pointed out the hydroquinone (HQ) as a harmful environmental pollutant able to aggravate experimental animal models that mimic RA. We postulate that a continuous pollutant exposure in low concentrations can alter the host homeostasis, weakening the individual ability to properly activate the immune system. Indeed, the HQ exposure did not cause any abrupt modification in the synovia or blood of healthy animals. Nevertheless, if the HQ exposure occurs during the sensitization and later CIA phases, it can definitely worsen symptoms and alter biochemical and histological parameters in CIA-rats [32,33]. The present study outlines HQ actions over human synoviocytes, leading them to proliferate, secrete cytokines, express TNF-α receptors, and produce ROS. Also, our data suggest that the activation of the AhR/IL-17 pathway is closely involved in the worsening of experimental arthritis in HQ-exposed mice.

In a clinical perspective, the critical role of synoviocytes on the progress of RA-related articular lesions led us to address the potential HQ mechanisms of action in these cells. Activated synoviocytes are known to secrete inflammatory mediators into the synovia, such as IL-6 and IL-8 [1,5], and remodel the extracellular matrix upon the production of structural components, including collagen and lubricating molecules [47]. Indeed, the higher frequency of CD90^+^ synoviocytes, the augmented deposition of collagen fibres in the synovia, and the elevated IL-6 levels of the synovial fluid from HQ-exposed CIA-rats indicate that FLS may be activated even upon an in vivo HQ exposure [32,33]. The inflammatory effects of HQ in healthy synoviocytes will be further investigated to determine the specific action of the xenobiotic in pre-stimulated cells.

RA human fibroblast-like synoviocytes (RAHFLS) have been widely employed to study the RA pathogenesis. Herein, we select them to evaluate the HQ influence over human cells. Indeed, the HQ exposure augmented the number of RAHFLS may be due to an enhanced proliferation rather than inhibition of cell death. HQ was previously described as an inducer of cell proliferation in the renal tubular epithelium as a mechanism to induce spontaneous progressive nephropathy and ensuing renal adenomas [48].

We have already displayed the HQ ability to activate TNF-α pathways in inflammatory process. In this context, the HQ exposure elicited the trachea reactivity mediated through a hyper TNF-α secretion by epithelial cells [29]. Also, our data clearly showed that the HQ exposure potentiates the TNF-α secretion and enhanced the expression of TNFR1 and TNFR2 in RAHFLS. Moreover, it has been fully demonstrated that the TNF-α interaction with TNFRs leads to the activation of NF-κB pathway, triggering the synthesis of inflammatory molecules [36,49,50]. We also showed here that TNFα-stimulated RAHFLS presented an increased expression of NF-κB p65, event unrelated to the HQ presence in the cell culture. Overall, these data corroborate previous studies that indicated a cell inability to upregulate NF-κB units (p65) under the HQ presence in vitro [51,52], unveiling that HQ may activate other signaling inflammatory pathways in synoviocytes.

To treat RA, an usual therapeutic approach attempts to block the TNF-α pathway, which ends up affecting a specific subset of regulatory T cells. TNFR2+ Tregs have been described to present a stable FoxP3 expression via gene hypomethylation [53,54]. However, TNFR2^−/−^ mice had an enhanced FoxP3 methylation in Treg cells and symptoms of a delayed-type hypersensitivity RA [54]. Also, selective agonists of TNFR2 were shown to augment the number of FoxP3 CD8+ T cells and alleviated CIA symptoms [53]. Therefore, the profile of the TNFR2 expression in other RA-related cells must be further investigated.

The RA regulation by AhR links the xenobiotics-stimulated immune system with the disease. This receptor is highly expressed in immune cells especially during the Th17 cell polarization, noted during the onset of some autoimmune diseases, including RA [21,22,24]. Some studies have indicated that AhR plays a role in exacerbating RA in smokers, especially due to IL-17A-derived actions [55,56,57]. Studies with experimental models have also suggested that AhR^−/−^ mice are more resistant to CIA and that AhR deficient-T cells can suppress the disease development [21]. Our previous studies demonstrated higher frequencies of AhR- and IL-17-expressing cells in the inflamed synovia of HQ exposed-rats, indicating that the AhR pathway could be activated by the HQ exposure in synovial cells [32,33]. Considering that AhR is expressed in RAHFLS and its activation results in cytokine secretion and cell proliferation [25,58], we investigated whether the HQ could exert its effects via AhR pathway. Whereas the HQ incubation enhanced the AhR activity in synoviocytes, the addition of an AhR antagonist reduced significantly the cell ability to proliferate and express TNFRs. Although our in vitro data with RAHFLS suggest that RA patients could display a similar pattern, experiments with primary synoviocytes derived from RA patients are still needed to confirm this possibility.

It is well-established that the AhR affinity and the degree of its activation by xenobiotics likely reflect the planarity, aromaticity and hydrophobicity of the ligand. Previous studies have shown that quinone structures display agonist activities to AhR. Nevertheless, the size of the hydrocarbon is important to fit within the AhR binding pocket. Therefore, it was suggested that aromatic hydrocarbons with one or two rings, such as benzene and HQ, could not activate AhR [59]. Indeed, benzene and HQ were not able to bind and activate AhR in Hepa1c1c7 cells [59]. Nonetheless, in vivo and in vitro studies contrast these data as follow: (1) benzene-mediating hematopoietic toxicity was not exhibited in AhR knockout mice due to, at least in part, the lack of AhR expression in bone marrow hematopoietic cells [60,61,62]; (2) benzene exposure up-regulated the AhR and Cyp1a1 expression in growth hormone producing-pituitary cells [63] and in primary pituitary cells of rats [64]; (3) benzene and HQ co-localized with AhR in Hepa1c1c7, without overexpressing Cyp2e1, a known marker of AhR activation, suggesting a non-classical pathway of AhR activation by benzene metabolites [65]. Although further studies are required to understand the controversial data about benzene and HQ as AhR ligands, our in vivo, in vitro and in silico data provide evidence that the AhR overexpression occurs after the HQ exposure in RA conditions. Furthermore, this pathway is associated with more severe symptoms of the disease in HQ-exposed mice.

As the HQ exposure was shown to enhance the oxidative stress in vivo and in vitro, it may affect the balance of pro-oxidant and anti-oxidant pathways. Considering that oxidative stress is a pivotal underlying mechanism for the RA progression [42,66], herein we confirmed the ensuing pro-oxidative HQ action in RAHFLS and suggested the involvement of this mechanism in the RA worsening. Nevertheless, the HQ-mediated oxidative stress was not reversed by the pre-incubation with an AhR antagonist, suggesting that HQ may also trigger toxic events in synoviocytes independently of AhR pathway.

In addition, the in vivo HQ exposure in AIA-C57BL/6 WT mice corroborated with the harmful effects of that xenobiotic on the development of experimental diseases that mimic human RA, as already demonstrated in CIA-rats [32,33]. Therefore, the HQ is an indubitable pollutant that worsens such as CIA as AIA experimental arthritis in different animal species. Animal models of inflammatory arthritis are extensively used to investigate the immunopathogenic mechanisms that culminate in inflammation-mediated articular damage. Among them, CIA and AIA are the two most common models utilized to mimic clinic symptoms of human RA [67]. However, each model features different mechanisms that drive the disease establishment. Whereas the CIA involves failure in the immunological tolerance, resulting in a systemic autoantibody-driven arthritis, AIA raises from an articular T cell-mediated damage and displays a detrimental pathophysiology [67,68]. Nevertheless, AhR-induced Th17 polarization is a fundamental pathway related to the enhanced inflammation in RA and to both experimental models [21,22,24,69].

Also, we showed here that the HQ exposure did not exacerbate the arthritis symptomatology in AhR- or IL-17R-KO mice, associating the AhR and IL-17 pathway with the HQ actions. In AhR and IL-17R-KO mice, RA-relating edema, pain, cytokines levels, synovial hyperplasia, influx of inflammatory cells and NET formation were reduced in HQ-exposed animals. Regarding the NET analysis, H3Cit has been described as a useful biomarker for early detection of NETosis [70], this process can occur in an independent H3 citrullination-manner as recently shown for atherosclerosis [71]. If that is the case for RA, it remains elusive. On the other hand, it has been demonstrated that the H3 citrullination can propagate the neutrophil activation [72], followed by the NET secretion. Also, an assay based on the quantification of NETs based on the detection of citrullinated histone H3 bound to DNA has been recently published [73]. Thus, our data indicate that the H3 citrullination staining detected in our study could indicate NET formation. Therefore, these data show a straight connection between AhR/IL-17 and HQ, whose actions may be further studied on diseases related to benzene or HQ exposures.

In conclusion, our data highlight the harmful direct effects of the HQ on cultured human synoviocytes, with the involvement of the AhR pathway. The AhR participation on the worsening of the HQ-driven RA was further corroborated with in vivo data that were linked to the activation of the IL-17 pathway. Hence, these data seem to contribute in clarifying the mechanism underlying immune-related diseases in smokers.

## Supplementary Materials

The following are available online at https://www.mdpi.com/article/10.3390/antiox10060929/s1. Figure S1: Viability of RAHFLS after the HQ treatment. Figure S2: Effects of the in vitro HQ exposure on the NF-kB activation in RAHFLS. Figure S3: In vivo HQ exposure evokes AhR expression on the synovia of CIA-rats. Figure S4: Effects of the in vitro HQ exposure on the expression of TNFR1 and TNFR2 in RAHFLS. Figure S5: AhR-Hydroquinone interaction mapping.
