# Supplementary material for: Hydroquinone Exposure Worsens Rheumatoid Arthritis through the Activation of the Aryl Hydrocarbon Receptor and Interleukin-17 Pathways
Source: Antioxidants (Basel). 2021 Jun 7;10(6):929. doi: 10.3390/antiox10060929 (PMC8229175; doi:10.3390/antiox10060929)
Supplement: Supplementary file 1 [file antioxidants-10-00929-s001.zip › antioxidants-1170438-supplementary.pdf]

Hydroquinone exposure worsens rheumatoid arthritis through the activation of the aryl hydrocarbon receptor and interleukin-17 pathways

Cintia Scucuglia Heluany[1]; Paula Barbim Donate[2]; Ayda Henriques Schneider[2]; André Luis Fabris[1]; Renan Augusto Gomes[3]; Isadora Maria Villas-Boas[4]; Denise Vilarinho Tambourgi[4]; Tarcilia Aparecida da Silva[5]; Gustavo Henrique Goulart Trossini[3]; Giovanna Nalesso[6]; Eduardo Lani Volpe Silveira[1]; Fernando Queiroz Cunha[2] and Sandra Helena Poliselli Farsky[1*]

Supplementary material:

Supplementary Data

Animals

The Animal Facility of the Faculty of Pharmaceutical Sciences and Chemistry Institute of the University of São Paulo supplied male 6-8-week-old Wistar rats. Animals were supplemented with food and water ad libitum. All procedures were performed according to the guidelines of the Brazilian Society of Science of Laboratory animals for the proper care and use of experimental animals. Experimental procedures were approved by the Ethics Committee on Animal Use (CEUA) of the University of São Paulo (protocol number 435). Before euthanasia, animals were anesthetized with a solution containing Ketamine/Xilazine 80:8 mg/kg, via i.p.

Collagen-induced arthritis (CIA)

CIA induction was performed as previously described [1]. Briefly, 2 mg/mL of bovine collagen type II was dissolved in 0.1 M acetic acid by gentle stirring overnight at 4 °C. The solution was emulsified in equal volumes of CFA. Whereas 200 μL of emulsified solution was injected via s.c at the base of the tail at day 7, only half of that volume was administered at day 14, through the same route.

In vivo hydroquinone (HQ) exposure

Animals were exposed to HQ solution at 25 ppm (1.5 mg/60 mL) through an ultrasonic nebulizer (NS®) for 1 hour/daily during 35 consecutive days as previously described (Heluany et al. 2018a,b). For comparison, control animals were exposed to saline or vehicle solution (5 % ethanol in saline) during the same period.

Anti-AhR Immunofluorescence analyses

Synovia derived from knee joints articulation of each experimental group (n = 4 animals) were collected, fixed in 10 % formalin and histologically processed. Tissues were embedded in paraffin and 5 μm sections were obtained. The slides were stained with an antibody anti-AhR (1:100), overnight. After 2 washing steps, they were incubated with a goat anti-mouse IgG Alexa Fluor 488 secondary antibody (diluted 1:400 in PBS + 0.1 % BSA + 0.01 % sodium azide), for 1 hour at room temperature. Slides were mounted with Vectashield containing DAPI and analyzed on a fluorescent imaging microscope (Imager.A2 Axio, Carl Zeiss). Images were acquired from 4 different fields per slide for each animal, totaling 16 fields investigated per group analysed with the Zen software (Carl Zeiss).

Cell-viability assay

A flow cytometry-based method using Annexin V and propidium iodide dye (PI) was employed to determine if HQ treatments could induce apoptosis or necrosis in RAHFLS. RAHFLS (1 × 10^4 cells/well) were seeded in 48-well plates. The cells were treated with different concentrations of HQ (1, 10 or 100 μM) for 24 hours. Thereafter, cells were washed with PBS, trypsinized, centrifuged (5 min, 1,500 g) and incubated with a FITC-conjugated Annexin V diluted in a binding buffer (1:100; BD Biosciences) for 30 min. After that, 150 μL of PI (1:400; Sigma-Aldrich) diluted in a binding buffer was added and 10,000 events were acquired in a flow cytometer (Accuri C6, BD Biosciences). The percentage of apoptotic (FITC positive and PI negative), necrotic (FITC negative and PI positive), late apoptotic (FITC and PI positive) and viable cells (FITC and PI negative) was quantified. Data are expressed as the percentage of viable cells, in apoptosis, late apoptosis or necrosis.

Detection of NF-kB activation in synovial cells

To evaluate the effects of treatments on NF-Kb activation, RAHFLS (1 x 10^4 cells/well) were plated in a 24-well plate and treated during 1 hour with HQ (1 or 10 μM) in the presence or absence of TNF-α (2 ng/mL) or only with TNF-α (2 ng/mL). Then cells were harvested and fixed with Cytofix/Perm buffer (BD Biosciences), permeabilized with Perm/Fix kit (BD Biosciences) and intracellularly stained for 1 hour at 37 °C with anti-NF-kB p65 primary antibody (1:100, Bioss). After two washing steps, cells were stained with a goat anti-rabbit IgG PE secondary antibody (1:200; Life Technologies), for 1 hour at room temperature. Then, 10,000 events were acquired by flow cytometry (Accuri C6, BD Biosciences). The analysis was carried out with the FlowJo software (Tree Star). Results are presented as mean fluorescence intensity (MFI).

AhR-Hydroquinone interaction mapping

To better understand the interaction points between AhR and HQ, hot spot generation and docking stimulation were performed. The AhR crystalized structure (PDB ID 4M4X) was submitted to FTMap [2] server to be screened by several molecular probes. The hot spots comprehended protein surface areas where the probes clustered. After determining the putative binding site, the protein structure from PDB was analysed. Then the AhR crystalized structure was submitted to energy minimization and preparation for docking simulations using AMBER99SB-ILDN force field [3] in GROMACS 5.1.4 software [4]. To simulate the physiologic conditions, water molecules (TIP3P model), NA+ and Cl- counter ions were added in enough quantities to the system and sequential rounds of energy minimization were performed until the total energy converged. Finally, the binding mode prediction was performed with DockThor [5], a docking server that employs the genetic algorithm for pose generation, and the polar and non-polar interactions were automatically identified using the Discovery Studio Visualizer (BIOVIA, Dassault Systèmes, Discovery Studio Visualizer).

References

1. Brand, D.D.; Latham, K.A.; Rosloniec, E.F. Collagen-induced arthritis. *Nat. Protoc.* **2007**, *2*, 1269–1275, doi:10.1038/nprot.2007.173.
2. Kozakov, D.; Grove, L.E.; Hall, D.R.; Bohnuud, T.; Mottarella, S.E.; Luo, L.; Xia, B.; Beglov, D.; Vajda, S. The FTMap family of web servers for determining and characterizing ligand-binding hot spots of proteins. *Nat. Protoc.* **2015**, *10*, 733–755, doi:10.1038/nprot.2015.043.
3. Lindorff-Larsen, K.; Piana, S.; Palmo, K.; Maragakis, P.; Klepeis, J.L.; Dror, R.O.; Shaw, D.E. Improved side-chain torsion potentials for the Amber ff99SB protein force filed. *Proteins Struct. Funct. Bioinform.* **2010**, *78*, 1950-1958.
4. Abraham, M.J.; Murtola, T.; Schulz, R.; Páll, S.; Smith, J.C.; Hess, B.; Lindahl, E. GROMACS: High performance molecular simulations through multi-level parallelism from laptops to supercomputers. *SoftwareX* **2015**, *1–2*, 19–25, doi:10.1016/j.softx.2015.06.001.

5. Santos, K. et al. Highly Flexible Ligand Docking: Benchmarking of the DockThor Program on the LEADS-PEP Protein–Peptide Data Set. *J. Chem. Inf. Model.* **2020**, *60*, 667–683.

Supplementary Figures

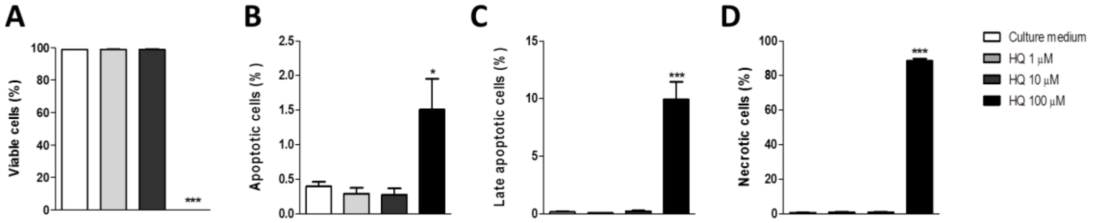

Figure S1: Viability of RAHFLS after the HQ treatment. RAHFLS (1 x 10^4 cells) were incubated with culture medium or with different concentrations of HQ (1, 10 or 100 µM) for 24 hours, and the cell viability (**A**), apoptosis (**B**), late apoptosis (**C**) and necrosis (**D**) were determine 24 hours later, through a flow cytometry-based method using Annexin V and propidium iodide dye. Data represent mean ± SEM from three independent experiments and were analyzed by one-way ANOVA. *p<0.05 and ***p<0.001 vs. all other treatments.

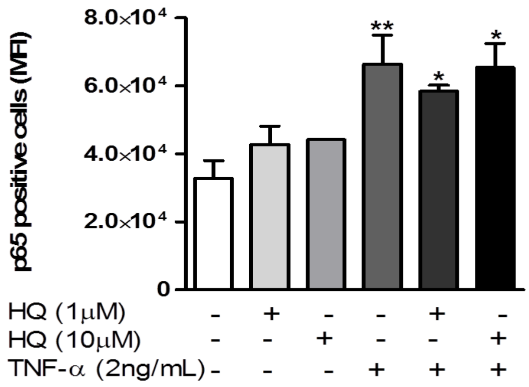

Figure S2: Effects of in vitro HQ exposure on the NF-kB activation in RAHFLS. RAHFLS (1 x 10^4 cells) were incubated with culture medium or with different concentrations of HQ (1 or 10 μM) in the presence or absence of TNF-*α* (2 ng/mL) for 1 hour. The NF-kB p65 activation triggered by synoviocytes was quantified by flow cytometry. Data represent mean ± SEM from three independent experiments and were analyzed by one-way ANOVA. *p<0.05 vs. respective groups treated only with HQ; **p<0.01 vs. culture medium.

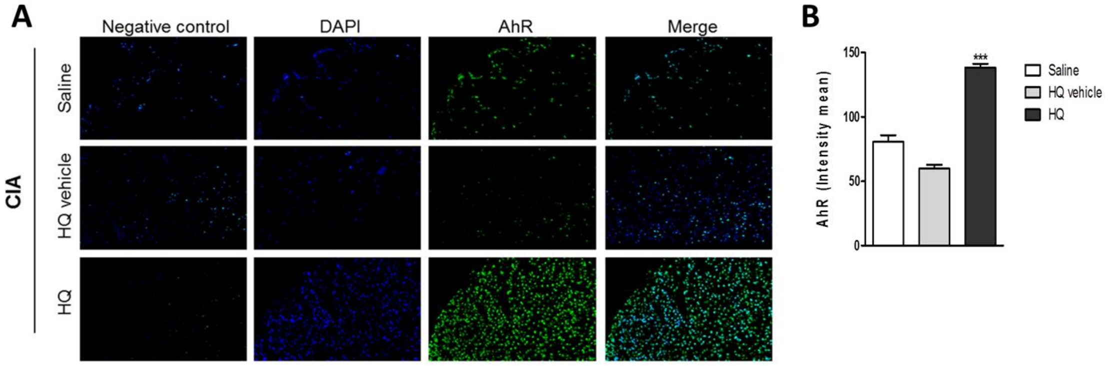

Figure S3: In vivo HQ exposure evokes AhR expression on the synovia of CIA-rats. Male Wistar rats were aerosol-exposed to saline, HQ vehicle (5 % ethanol in saline) or HQ (25 ppm) for 1 hour, daily, by nebulization, from day 1 to 35. Bovine collagen type II (0.4 mg/200 μL) was injected into the subcutaneous tissue of the base of the tail on the 7th day and a booster injection (0.2 mg/100 μL) was carried out seven days later by the same route On the 35th day of exposures, synovial membranes from CIA-exposed rats were collected and analyzed by immunofluorescence technique for AhR-labeled cells (**A**, **B**). DAPI – positive staining for nuclei. Original magnification - 20x. Data represent mean ± SEM of four rats in each group and were analyzed by one-way ANOVA. B: ***p<0.001 vs. saline and HQ vehicle CIA groups.

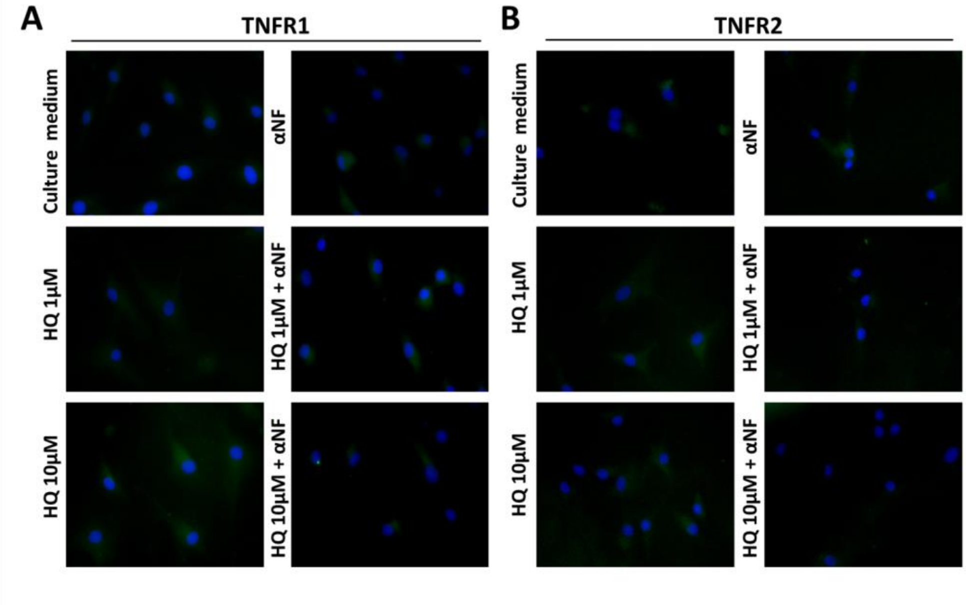

Figure S4: Effects of the in vitro HQ exposure on the expression of TNFR1 and TNFR2 expression in RAHFLS. RAHFLS (1 x 10^4 cells) were incubated with culture medium or with different concentrations of HQ (1 or 10 μM) in presence or absence of the AhR antagonist α-naphthoflavone (αNF, 100 μM) for 24 hours and the expression of TNFR1 (**A**) and TNFR2 (**B**) were quantified by an immunofluorescence assay and the representative images are presented. DAPI – positive staining for nuclei. Original magnification – 40x.

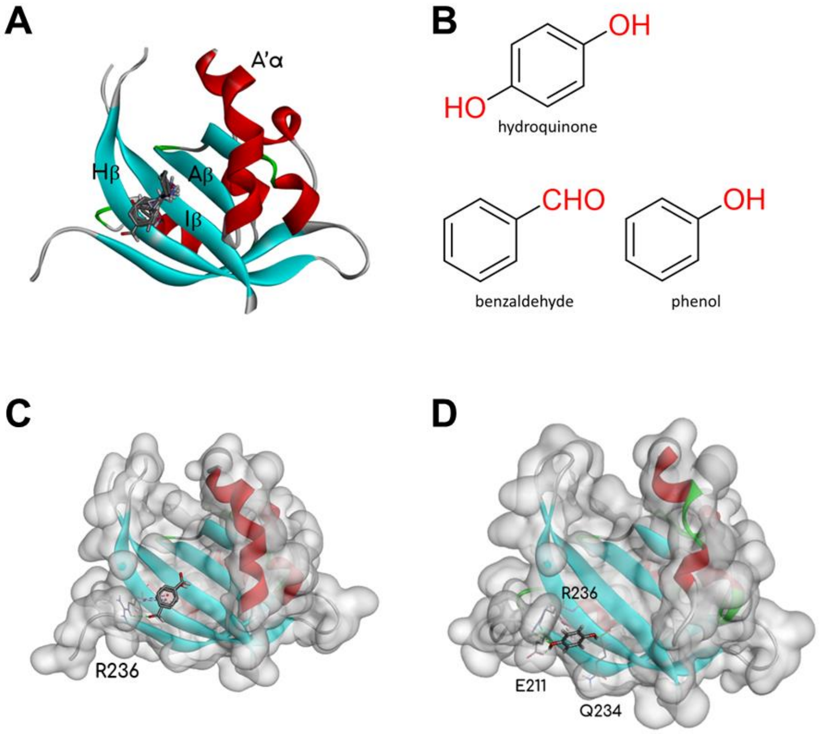

Figure S5: AhR-Hydroquinone interaction mapping. To better understand and confirm the interaction between HQ and AhR, we performed an in-silico approach. (A) AhR crystalised structure (PDB ID 4M4X) with clustered FTMap probes and labelled secondary structures. (B) 2D structures of HQ and probes that share similar structural features, with hydrogen bond acceptor groups highlighted in red. (C) Phenol and benzaldehyde probes interacting with R236 via polar/non-polar contacts. (D) Docking-predicted hydroquinone binding mode in the AhR structure. Protein structure in cartoon (α-helixes represented in red and β-sheets in blue) and additional surface in C and D, and the ligands were represented in sticks.
